# Supplementary material for: Are carbon emissions trading and green financial instruments synergistic? -Comprehensive quantitative research based on content analysis
Source: PLoS One. 2024 Mar 7;19(3):e0298601. doi: 10.1371/journal.pone.0298601 (PMC10919601; doi:10.1371/journal.pone.0298601)
Supplement: S1 Data — (ZIP) [file pone.0298601.s001.zip › Data/地级市Z_F工作报告.zip!/╔╜╬≈/╜·╓╨╩╨/└┤╫╘╣1⁄2╓┌║┼í╛┬φ┐╦╩2╛▌═°í┐_2019.docx]

各位代表：

现在，我代表市人民政府向大会报告工作，请予审议，并请市政协委员和列席人员提出宝贵意见。

一、2018年工作回顾

2018年是晋中发展卓有成效的一年。在省委、省政府和市委的坚强领导下，市政府坚持以习近平新时代中国特色社会主义思想为指导，全面、准确、创造性地贯彻落实习近平总书记视察山西重要讲话精神和中央、省、市一系列大政方针、决策部署，坚持“三个三”工作思路和举措，锐意改革、狠抓落实，以钉钉子的精神锲而不舍推进各项工作，经济社会发展取得新突破。

我们抢抓发展机遇，推动政策措施落地落实，经济运行稳中向好。坚持压实责任抓落实，分解落实政府系统“三个清单”任务147项，以上率下、督办销号、一抓到底。坚持用足政策抓落实，把贯彻落实上级方针政策和决策部署作为推动发展的主要任务，深刻领会、跟踪对接、用活做实，促进了问题解决、任务推进。坚持问题导向抓落实，开展“激活农村资源、促进乡村振兴”攻坚行动，出台支持山西农谷建设、民营经济发展等政策措施等，有效破解了发展难题。坚持尽锐出战抓落实，围绕脱贫攻坚精准注入50多亿资金，1079个工作队3384名队员奋战在脱贫一线；围绕环保攻坚首创市级环保督察、约谈和空气质量改善奖惩，环保执法共行政处罚1309户、罚款1.4亿。坚持加大投入抓落实，市城区城建重点工程当年投资190亿，规模体量创历史之最。在各项任务落地落实中，全市经济社会发展呈现稳中有进、进中向好的势头：经济增速持续回升，完成地区生产总值1447.6亿，增长7.1%；规模以上工业增加值566.3亿，增长8%；社会消费品零售总额656.3亿，增长8.1%。发展效益持续向好，一般公共预算收入150.8亿，增长27.7%，增速为近12年最高；规模以上工业企业主营业务收入、利税额增长20.8%、33.7%，均高于全省9.4个百分点；城镇和农村常住居民人均可支配收入完成3.3、1.3万元。转型升级持续夯实，固定资产投资702.7亿元，增长9.6%，转型项目投资占64.4%；招商引资签约项目当年开工率63.6%，高于全省3.6个百分点。

我们咬定转型项目，加快新旧动能转换，转型发展引擎强劲。深入开展“转型项目建设年”活动，健全完善逐月调度、及时开工、每季企业家项目对接、半年政银企对接、常态化帮扶、产业链招商等机制，促进了新动能成长和产业结构优化升级。龙头优势凸显，安泰集团、吉利汽车产值分别达164和131亿元，东方希望铝业、中煤晋中能化等向百亿企业迈进；底特律电动跑车、金科山西智慧科技城、苏宁山西电子商务中心等世界和国内500强企业项目落地实施。新产业带动增强，新能源汽车快速成长，旅游总收入过千亿，装备制造、战略性新兴产业、高技术产业、非煤产业增加值分别增长20.2%、54.6%、5.9%、14.2%，三产对经济增长贡献率56.9%。开发区成效初现，晋中开发区10个特色产业园区、5大产业集群集聚发力，“1+10”联合体建设稳步推进，省级及以上开发区达到9家，全年固投完成266.8亿元，比上年增长72%，贡献了65%的新兴产业产值。能源革命步伐加快，关闭淘汰2座矿井，退出年产能54万吨，煤炭先进产能达39%；风电、光伏发电总装机容量分别达到172.2万千瓦和81万千瓦，寿阳光伏全产业链示范区、灵石煤电铝材一体化循环示范园积极推进，“先行区”基础进一步夯实。创新创业蓬勃发展，全市新增各类市场主体4.1万户，高新技术企业141户，省级以上重点实验室、工程（技术）研究中心、众创空间达45家。

我们坚持“三农”优先，突出山西农谷示范引领，乡村振兴全面开局。高标准出台《实施乡村振兴战略总体规划》和市县乡村户“五级示范”标准，以点带面推进乡村振兴。农谷引领深度融合，围绕科技创新和深化改革，“十方面突破”的62项年度任务全面完成，总投资286亿的63个重点项目全面实施，国家现代农业产业科技创新中心等7个国家级试点项目顺利落地。承前启后、影响深远的全国农村改革（太谷）论坛成功举办，国家级农高区升建通过专家评审。构建“1+10+X”融合发展体系，形成全市域协同联动新格局。农业结构调优提质，形成太谷国家级、灵石省级和9个市级产业园格局，特色农业占比达75%；榆社十四只绵羊生态养殖基地、太谷番茄小镇等一批龙头项目，带动全市农业向专业化、集约化、规模化快进，全市农产品加工业销售收入237亿元。农村环境明显改善，全市658村垃圾得到无害化处理，222村具备污水处理能力，改造农村公厕、户厕4.6万座，创建118个省市级美丽宜居示范村，承办了全省乡村振兴暨农村人居环境现场会。

我们聚力城市升级，大力推进建管提质，宜居宜业现代化城市迈出大步。把做好城市工作作为满足人民群众美好生活向往的重要支撑，同步推进规划、建设、管理，统筹生产、生活、生态，城市承载力、影响力、发展力突破性提升。基础建设大提速，环城道路、龙城东延、龙湖东延以及安宁街、迎宾街改造等8条干道完工，榆祁高速龙湖收费站建成通车，15条小街巷完成改造，外环快速路骨架形成，微循环进一步畅通；市民关注的工人文化宫、晋中大医院、“三馆”等投用；保障性安居工程完成1.5万套。城市品位大提升，强化一半森林一半城的建设理念，生态绿廊、顺城公园基本建成，新增绿地119.8万平方米，基本实现300米见绿、500米见园，潇河18.5公里生态修复治理成效显现；12段黑臭水体整治完成，市城区禁燃区扩大二倍，垃圾发电项目实质性启动，空气质量综合指数好转率、SO2均值降幅、二级以上天数同比增加数均排全省第一。城市框架大拓展，高标准启动龙城大街区域开发，高起点规划实施万亩职教城，强力度推动示范区潇河产业园起步建设，晋中太原一体化发展步入新阶段。

我们强化为民宗旨，积极呼应群众期盼和向往，人民美好生活开启新篇。把群众的小事当作政府的大事，一件接着一件办，民生支出占到一般公共预算支出的82.4%。脱贫攻坚连战连胜，产业就业发挥治本作用带动3.3万人稳定脱贫，易地扶贫搬迁完成158个整村和2.6万人，贫困村基础设施公共服务历史欠账基本补齐，全市6.8万贫困人口脱贫、204个贫困村退出，和顺、左权脱贫摘帽指标全部达标。社会事业全面进步，成功创建国家公共文化服务体系示范区，市级“五馆一院”和县级“两馆一院”全部建成并免费开放，全民健身活动广泛开展；541所办学主体学校完成校长职级制改革，新建、改（扩）建公办幼儿园30个；县乡医疗卫生机构一体化步伐加快，家庭医生签约服务提质增效。社会保障更趋完善，生育和医疗保险合并实施试点通过验收评估，机关事业单位养老保险入轨运行，城镇新增就业、失业人员再就业完成5.06万和1.08万人。安全生产稳定向好，实现各类生产安全亡人事故总起数和死亡总人数等“八个双下降”。政府承诺的全市行政村全部通客车、无线网络全覆盖、县乡农村物流节点全覆盖等十件民生实事如期兑现。

我们秉持打铁自硬，努力提高依法施政能力和服务水平，政府自身建设全面加强。坚持依法施治，认真执行市人大及其常委会决议决定，主动报告工作和重大事项，依法向市人大常委会提请审议两部地方性法规草案；自觉接受人大、政协监督，依法办理市人大代表建议354件、市政协委员提案372件。加速权力“瘦身”，企业投资项目承诺制改革实施项目216个，受到国务院和省委、省政府肯定，市级政府投资项目备案公示制改革启动，“一网十平台”完成了四梁八柱，实现市县政务服务“一张网”,“网上办”事项达85%，“马上办”事项87%、“政务闪办”事项20%、“零跑腿”事项35%，新开办企业“照证章税户”1天办好。严格落实全面从严治党主体责任，深入推进反腐败斗争，源头遏制“四风”，市政府常务会听取巡察工作情况汇报，积极推动审计发现问题整改，动态掌握政府系统违纪违规问题线索，持续“六大纪律”利剑高悬，巩固提升了风清气正、担当作为的干事创业良好局面，努力实现海晏河清。

一年来，民族、宗教、外事、侨务、港澳、对台、援疆、统计、档案、史志、社科研究、科普、气象、地震等工作取得新进展，妇女、儿童、青少年、老龄、残疾人、红十字、慈善等事业又有新进步，人民防空、双拥、国防后备力量建设等扎实推进，社会和谐稳定。

各位代表，我们以奋斗的姿态走过2018，高质量转型发展的路子越走越宽。这是习近平新时代中国特色社会主义思想和习近平总书记视察山西重要讲话精神在晋中落地生根的结果，是省委、省政府正确领导，市委统揽全局，市人大、市政协监督支持的结果，是全市上下播撒汗水、共同拼搏的结果。我代表市政府，向全市广大干部群众，向各位代表、各位委员，向各民主党派、无党派人士、工商联、人民团体和社会各界人士，向驻地人民解放军、武警官兵、公安民警和国家、省驻地单位，向所有关心支持晋中改革发展的朋友们，表示崇高敬意和衷心感谢！

回顾一年奋斗历程，我们深刻体会到，办好晋中的事情，必须始终保持绝对过硬的政治定力，牢固树立“四个意识”，忠诚践行“两个维护”，坚决贯彻中央、省大政方针，结合晋中实际创造性落地落实；必须始终保持高质量转型发展的战略方向，把握晋中独特的区位优势和发展机遇，不断培育壮大经济发展新动能；必须始终发扬直面问题矛盾的斗争精神，把解决问题、狠抓落实作为政府工作的天职，以敢于斗争的勇气、善于斗争的本领，拓开新局面、创造新优势；必须始终坚持以人民为中心的发展思想，聚焦群众关切，呼则必应、谋则必举、干则必成，让发展成果更多转变为全市人民的实惠。

各位代表，船到中游浪更急，人到半山路更陡。我们清醒地认识到，晋中发展仍面临不少困难和挑战。从外部看，我国经济在保持总体平稳、稳中有进的同时，也出现稳中有变、变中有忧的新趋势，风险挑战增多、困难压力加大。从内部看，我市长期积累的结构性、体制性、素质性矛盾远未解决，煤炭占比依然较高，新兴产业支撑能力依然不足，环保约束压力依然较大；一些领导干部不敢为、不会为、不作为还不同程度存在，等等。这些都需要我们坚持底线思维，持续苦干实干，认真加以解决。

二、2019年工作安排

2019年是新中国成立70周年，是全面建成小康社会关键之年，是我市推进高质量转型发展的攻坚之年。全省正处于在“两转”基础上拓展新局面的关键期，我市重大发展机遇叠加,转型动能加速集聚，我们要以竞进“赶考”的姿态，持续走好晋中高质量转型发展之路。做好政府工作，要以习近平新时代中国特色社会主义思想为指导，全面贯彻落实党的十九大精神和中央、省、市一系列重大决策部署，坚持稳中求进工作总基调，坚持新发展理念，坚持推动高质量发展，坚持以供改和综改相结合为主线，坚持深化市场化改革、扩大高水平开放，围绕决战转型综改主战场、争创乡村振兴示范市、建设能源革命先行区、打造创新创业新高地“四个目标”，运用“三个三”工作思路和举措，增强担当作为的主动性、贯彻落实的创造性和改革发展的实效性，不断提高经济发展的“含金量”“含新量”“含绿量”，决胜全面挺进全省第一方阵，决战全面建成小康社会，率先走出具有晋中特色的资源型经济转型发展新路，增强人民群众获得感、幸福感、安全感，以优异成绩迎接中华人民共和国成立70周年。

综合考虑发展所需和转型导向，建议全市经济社会发展主要预期指标为：地区生产总值增长6.3%左右，全社会固定资产投资增长7.5%，规模以上工业增加值增长5%，社会消费品零售总额增长7.5%，一般公共预算收入增长7%，城乡居民人均可支配收入分别增长6.5%和7%以上，居民消费价格涨幅控制在3%左右，城镇新增就业3.7万人，城镇登记失业率控制在4.2%以内。资源节约、环境质量和民生改善方面的约束性指标不折不扣完成省下达任务。

围绕上述目标，重点抓好八方面工作：

（一）抓好转型项目建设，着力推动产业结构优化升级。今年是全省“深化转型项目建设年”，要着力实施好当年投资653亿的437个亿元以上重大项目，引导新兴产业强链条、壮集群，传统产业优结构、提层级，服务行业提质量、促融合。

一要以千亿新能源汽车产业基地为龙头，培育壮大制造业集群。按照龙头带动、链式布局、园区承载、集群发展的思路，加快培育百亿企业、千亿产业。以推动吉利、底特律、福坛三个汽车品牌集聚为主抓手，着力推进零部件配套和锂电池生产共享，重点实施底特律电动跑车、福坛车桥、斯纳德专用车等7个项目，加快形成年产整车30万台、核心零部件2000万台（套）、动力电池40GWh的千亿产业集群，同时推进左权东方希望铝、山西建筑构件和航空产业园、昔阳锂离子电池材料等项目建设。优化特色食品产业，实施北方功能食品产业园、海玉食品扩产、红星六曲香10万吨原酒生产等项目，形成山西中部食品工业集聚区。

二要以煤炭、焦化重组改造为重点，推动能源革命纵深发展。抓住我省开展能源革命综合试点机遇，持续推进煤炭减量重组，力争已报方案获批；加快自动化、智能化煤矿建设，大力发展先进产能，推动产业结构优化升级。加快改造提升焦化产业，实施好介休路鑫、昌盛茂胜两个500万吨级，灵石聚源、聚义两个300万吨级大机焦及化产项目。加快寿阳光伏发电全容量并网和昔阳增量配网+智慧农业产业园落地，积极推进已批准的10块煤层气区块开发利用。抓住我省解决火电历史遗留问题机遇，争取阳煤寿阳明泰、华能左权2个低热值煤发电项目尽快复工，灵石启光、介休崇光低热值煤发电项目双机投产和民生供热。

三要以全域旅游示范区建设为突破，大力发展民生内需产业。围绕创建国家全域旅游示范区，统筹文化文物事业和文化旅游产业发展，宜融则融、能融尽融，以文促旅、以旅彰文。重点做优做强5A景区，抓好平遥古城基础设施改造提升、乔家大院综合开发、王家大院创5A等项目，大寨景区、榆次老城努力向5A水平升级；发展文旅新业态，实施好潇河“一条玉带、八颗明珠、三大方舟、十个部落”和太谷乐华欢乐度假区、榆社云竹湖、左权百里画廊、榆次黄土文化雕塑园、祁县古城开发等项目，同步抓好专线公路、集散中心、旅游厕所、房车营地等配套设施；做大做实晋中文化旅游与工业农业、体育教育、健康养老、庄园田园等领域深度融合，支持企业转型、乡村振兴、中医中药、节展赛事等业态参与进入文化旅游；加强文化文物遗产保护利用，进一步深化文保景区景点“两权分离”体制机制改革，鼓励文创产品开发，扩大文旅融资平台，用创新激活资源，用资源换取资本，加快把文旅产业培育成战略性支柱产业和高质量幸福产业。持续推动广誉远中医药小镇、黄河制药等项目，进一步弘扬经典国药。积极培育健康养老家政、教育培训托幼等服务消费，增强消费拉动。

各位代表，抓好转型项目，晋中高质量发展才有底气。我们将牢固树立服务项目就是服务转型的理念，积极围绕转型项目建设各要素、各环节做好对接服务，重点落实好企业投资项目承诺制和政府投资项目公示备案制，以项目建设为转型发展做实硬支撑，获得高效益！

（二）抓好百强千企万户培育，着力推动民营经济高质量发展。着力落实支持民营经济发展“省30条”“市25条”，实施“百强千企万户”培育工程，用三年时间集中扶持百强行业领军企业，帮扶千户“四上”重点企业，新培育万户小微企业，促进实体经济入规进限。

一要真金白银扶持发展。通过降低成本、增加投入，为民营企业提供80亿的资金支持。市级财政安排6亿元资金，可撬动社会资本30亿元以上：将企业应急周转保障资金由1亿元增加到3亿元；将政府性融资担保机构注册资本增加5000万元，对融资担保费率低于3%的差额部分进行补贴；市级中小企业发展资金增加到2000万元，用于担保公司注入、金融机构奖励等；安排电子商务发展专项资金1000万元，优先支持具有一定规模的民营电商；设立1000万元小微企业信贷风险补偿金，促进银行加大信贷投放；设立3000万元人才发展专项资金，支持民营企业引进高层次人才；鼓励和支持有条件的企业开展信用评级，降低融资成本。用足用好3000万元科技专项资金，研究制定科技计划项目评审办法和资金扶持标准，落实研发投入、高新技术企业、专利、科技成果奖励等政策措施，推动由“事前资助”向“事后补助为主、事前资助为辅”转变，力争各类创新载体平台达到50家以上。激励质量、品牌和标准化建设，对晋中品牌创新成果、精益管理优秀企业、质量攻关重点项目、标准创新及标准化试点示范项目给予2-30万元补助。

二要真心实意扶持服务。市里每两年发布百强行业领军企业名单，并建立动态调整机制，对进入全国民企500强的，市级财政再奖励100万元。实施新晋商企业家素质提升工程，3年内实现重点民营企业董事长或总经理教育培训全覆盖。依法保护企业家人身和财产安全，妥善处理历史形成的产权纠纷和民营企业涉纪涉法问题，大张旗鼓树立民营企业家典型，让企业家切身感受到社会的信任和尊重。

三要真抓实干解决问题。一是开放投资领域，实施市场准入负面清单制度，实现“非禁即入”，鼓励民间资本投入基础设施、生态环保、脱贫攻坚、教育医疗等领域，引导和促进民间资本参与PPP项目，支持参与国企混改、军民融合发展。二是完善帮扶服务机制，每名市级领导联系3户“百强行业领军企业”，县级领导对千户“四上”重点企业帮扶全覆盖。三是加大要素保障力度，对新兴产业、优势产业且用地集约的工业项目，土地出让底价可按不低于本地土地等别的70%执行，最低出让年限放宽至30年，最高50年；开发区在已建成标准厂房200万平米基础上再建100万平米。

各位代表，晋中的发展，民营经济居功至伟，民营企业家功不可没。我们要大力弘扬晋商精神，聚焦实业、主业，通过让企业获得实实在在的政策，推动新晋商勇立潮头！

（三）抓好山西农谷辐射引领，着力推动乡村振兴迈出实质性步伐。坚持农业农村优先发展，统筹实施好乡村产业振兴、美丽乡村建设、乡村文化兴盛、乡村现代治理、乡村民生提升、体制机制创新、基层组织建设七大行动，为全面建成小康社会打下坚实基础。

一要发挥山西农谷示范引领作用。紧紧抓住升建国家级农高区契机，围绕科技创新和深化改革，在平台打造、项目建设、辐射带动等方面取得新突破。促进谷城院一体化发展，抓好太谷国家现代农业产业科技创新中心和国家功能杂粮技术创新中心等“四院八中心”建设，引进食品产业技术研究院，推动建设省级农业标准化技术委员会，年内建成2-3个协同创新平台，形成3-5个科技成果转化。推进华为农业大数据中心、番茄小镇、大北农绿色种养循环、南方食品两岸产业园等标杆项目建设，做好先正达农药种子平台，做活山西省农产品交易中心，力争月月有活动。加强山西农谷与各县“飞地经济”深度合作，拓展“1+10+X”融合发展新模式，形成一批对接成果，让农谷红利惠及全市。

各位代表，山西农谷建设是省级战略，正在创建国家农高区，我们将以更高标准、更高层次建设研发高地和产业洼地，努力形成乡村振兴的样板，为全省作出示范！

二要推动产业提质增效。坚持质量兴农、绿色兴农，稳步提升粮食生产能力和畜、果、菜品质。大力发展有机旱作农业，实施高标准农田建设、秸秆还田地力培肥、耐旱技术集成推广、标准化试点示范等工程，建立独具特色的有机旱作农业体系，争取全国有机旱作农业现场会在我市召开。抓好11个农业产业园建设，着力培育新型经营主体，规范提升示范合作社和示范家庭农场，积极推动全国龙头企业山西谷瑞达农产品交易市场在榆次、太谷落地建设，尽快形成国内外农产品集散地。

三要深化农业农村改革。继续办好全国农村改革（太谷）论坛，用好理论实践成果。进一步完善农村承包地“三权分置”改革，健全农村产权交易体系，有序引导土地经营权流转和适度规模经营。认真落实扶持服务小农户政策措施，促进小农户和现代农业发展有机衔接。不断完善双层经营体制，积极推动集体经济组织成员身份确认全面完成，深入推进农村集体产权制度改革。推广寿阳林权改革经验，扩大对非林地经济林木（果）权颁证、资产评估、抵押贷款。深化供销合作社综合改革，搭建综合服务平台。扎实推进“激活农村资源、促进乡村振兴”攻坚行动，把发展生态庄园经济和农林牧PPP项目作为主抓手，让各类经济实体和现代职业农民成为农业发展主力军，吸引城市资本、人才、技术“上山下乡”，使资源盘活、升级、增效，培育“三农”发展新动能。推进中国职业农民大学筹办，积极开展新型职业农民培训，实施认定和职称评审，培训新型职业农民2万人，让党的农村政策的阳光雨露惠及晋中大地。

四要提升乡村治理水平。深入学习浙江“千村示范、万村整治工程”经验，重点做好垃圾污水处理、厕所革命、村容村貌提升等工作，打造20个示范乡镇、100个示范村和1万个示范户。强化五级书记抓乡村振兴制度保障，夯实基层党组织建设，建立健全党组织领导的自治、法治、德治相结合的乡村治理体系，高标准打造100个社会主义核心价值观示范点，积极推进文明乡镇、文明村创建。

（四）抓好市场化改革和高水平开放，着力推动转型发展动力活力持续增强。深化对改革开放是重要法宝、必由之路、关键一招的认识，坚持“七个紧紧抓住”推进改革思路，以改革再深化激活发展机制，以开放再扩大拓展发展空间。

一要抓好重点领域改革。破题综改试验区建设。重点发挥晋中开发区引领作用，以开发区为载体，建设产业创新平台，打造金融服务体系，培育重点转型企业，打造数字政府，推进开发区全价值链一体化发展，率先构建新兴产业创新生态系统，加快晋中全市域转型综改试验区建设。继续加大省级开发区申报力度，着力提升开发区“三化三制”水平，重点打造专业化队伍，在国际化方面实现破题。深化国资国企改革。有序推动国有资本授权经营体制改革，组建市国有资本投资运营公司；创新破产、僵尸国有企业市场化退出机制，组建市国企资源托管服务有限公司。有效推动中央、省属国有企业“三供一业”改造工程，力争年内完工。落实财税金融体制改革任务。实施好基本公共服务领域市县财政事权和支出责任划分改革，加强预算绩效管理，提高依法用财、科学理财水平；深入开展社会保险费和非税收入征管职责划转；加强政府债务管理，积极争取地方政府债券，坚决制止违法违规举债行为。做好农信社改制化险扫尾工作，运行好区域金融风险监测平台，保持打击非法集资高压态势，有效防控金融风险。

二要提升对外开放水平。对接“一带一路”、京津冀协同发展、雄安新区建设等重大战略，主动“走出去”“融进去”，切实“引进来”“留得住”，发展更高层次的开放型经济。重点实施好外贸主体培育工程，为全市出口企业提供政策培训、业务指导、市场开拓等服务；实施出口品牌培育工程，利用外经贸扶持资金支持企业开展国际品牌认证，做优做强玛钢、碳素、玻璃器皿等出口品牌；实施国际营销网络建设工程，支持“海外仓”等境外营销网点，开展“千企百展”活动；实施外贸新业态培育工程，支持跨境电商和外贸综合服务企业发展，创建“一带一路”中小企业特色产业合作区。

三要提高招商引资质量。把招商引资、招才引智作为衡量对外开放成效的重要标尺，继续强化各级一把手抓招商引资责任，聚焦我市重点发展的主导产业，细化各县（区、市）、各开发区的重点产业和项目，精准开展以建链、补链、强链为主的产业链招商，重点对标先进、对接龙头，瞄准世界500强和国内领军型、科技型、品牌型企业招商，吸引一批高科技高品位项目落地。

（五）抓好城建重点工程建设，着力推动区域协调发展。省委省政府提出，“要把山西中部盆地城市群一体化作为重大战略强力推进”“要推动太原晋中一体化发展”，为晋中城市建设和区域协调发展带来新的机遇。我们要突出市城区产业集群、人口集聚、功能辐射，加快形成以市带县、协调互动的良好发展格局。

一要高起点建设新区。积极对接太原都市区，推动市城区向北、向西扩张，多方面承接太原产业、人口转移，城市框架由100平方公里扩大到150多平方公里。北部，启动龙城大街区域开发，以龙城大街为轴，以美好生活为主题，布局冰雪运动、体育产业、旅游集散、房地产开发等业态，打造“一轴一环三带多组团”，与太原龙城大街形成40华里经济长廊，成为三年总投资过千亿、迈万亿的投资热区，晋中城市转型升级的特色新区。南部，在高标准完成潇河城区段综合整治的基础上，实施108桥至郝村段12公里治理工程，形成与榆次老城、爱琴海购物公园互动，与太谷县改区呼应的60华里产城融合洼地。东部，规划建设万亩职教城，整合晋中职业院校入驻，积极引进省内外优质职教资源，深度推进产教融合、校企合作，打造引领辐射全省的产教融合示范区。西部，着力推进晋中开发区建设，加快杨盘街、安宁街、机场东路等道路与太原对接，积极推动轨道交通建设。

二要高标准完善配套。一是完善交通网络。重点构建主框架，东环北延与龙城大街衔接，形成通往省城的又一快速通道；小牛线与108国道、太太路相接，成为东西向快速通道；农谷大道全线通车，成为太原、榆次、太谷的快速干道。补齐大网络，5月底前完成南环西延扫尾，与西环路、农谷大道贯通；锦纶路南延至南外环，实现城市内外快速路闭环。畅通小循环，顺城街西延打通与晋中高铁站通行瓶颈，广安街由环城东路连接至吉利路，府东路北延至蕴华街；实施8条小街巷改造，让群众出行更便捷。二是完善服务设施。加快广安中学、经纬中学、实验小学等建设，启动市传染病医院、妇幼保健院、第四人民医院迁建。实施好苏宁易购、田森商业壹中心等大型商业综合体，推进便民市场华联模式。三是加快保障性安居工程建设。加大城中村、城镇棚户区改造力度，完善住房租赁市场，满足人民群众多样性住房需求。

三要高效能强化管理。突出市场化、专业化、精细化、智能化，像绣花一样管理城市。狠抓城市顽疾治理，推进电线下地、广告下墙、汽车进库，解决好交通拥堵、乱停车、“马路拉链”等突出问题。推动城市智管、细管、众管，在全市全面推行城管执法“三勤合一”新模式，配套建设市县一体数字化城管平台和指挥体系，打造多元共治、良性互动的城市治理新模式。持续抓好“四城联创”，市城区创建国家园林城市，推进国家卫生城市创建，平遥、祁县、太谷、榆社、和顺、寿阳六县和13个乡镇创建国家卫生县城（乡镇），努力实现国家卫生县城全覆盖。

四要高层次统筹城乡。以完善基础设施网络为抓手，积极构建中心城市和小城镇协调发展格局。今年开工昔阳至榆次高速、208国道清徐至祁县段建设等，完成1300公里四好农村路建设。改善电力设施，启动三年投资30多亿元、新建改造近200公里供电线路、低压线路的电力工程。加快5G商用步伐，力争在二青会场馆、大型商场、重点高校等区域建立5G试点基站。加快建设产业、生态和功能特征鲜明的特色小镇和特色小城镇，进一步丰富内涵、提升质量。

（六）抓好深度贫困人口脱贫，着力推动脱贫攻坚决战决胜。在巩固已摘帽和即将摘帽县脱贫成果，推动非贫困县升温加压的同时，聚焦深度贫困，高质量完成1.7万人62个贫困村脱贫退出、榆社县脱贫摘帽任务。

一要主攻榆社深度贫困。举全市之力支持榆社，市级出台专门意见，统筹省市资金5.2亿支持榆社脱贫摘帽，并充实市直帮扶单位；榆次、介休、灵石、寿阳和开发区按照不低于一般预算收入1%的资金连续两年对口援助，其他县暂不实施的项目资金、用地指标优先调剂榆社使用。榆社县要紧紧扭住产业扶贫和易地搬迁两大重点不动摇，推动“四种四养”主导产业扩量提质增效，确保7月底前事关整县脱贫的重点工作全部完成，年底前14项指标全部达标。

二要狠抓产业精准扶贫。持续引进和培育壮大农业龙头企业，不断提升对贫困人口的覆盖面和带动力；积极完善带贫益贫机制，使全市60%以上的贫困人口都能靠龙头企业带动和专业合作社吸纳发展产业、稳定脱贫。大力夯实脱贫产业硬支撑，在巩固完善去年各县完成的特色农业、旅游、光伏、电商等914个扶贫项目基础上，今年要落实好总投资8.8亿元的1100个特色产业项目，特别要持续抓好易地扶贫搬迁后续产业，为搬迁群众增收提供厚实支撑。

三要筑牢特殊群体防线。紧盯因病、残、灾和年龄等因素，家庭主要劳动力失能的深度贫困群体，持续筑牢政策保障防线。对已脱贫的要建立返贫预警机制，全面推广返贫责任保险；未脱贫的要落实好“双签约”“三保险三救助”、义务教育“两免一补”等政策，实现社会保险全覆盖。高度关注收入在贫困线边缘的非贫困人口，探索差异化支持政策和保障措施，在政策框架内扩大农村低保、社会救助覆盖面；对无劳动能力的要通过入股分红、公益岗位、生产奖补等形式，开辟政策兜底之外的收入渠道。

四要力推内生动力提升。进一步转变扶贫方式，坚持落实帮扶政策与贫困群众参与相挂钩，引导贫困群众通过劳动获得收入。把贫困村创业致富带头人培育作为激发内生动力的长效之举，对村两委成员、新兴经营主体负责人、返乡能人等实施分类培训，坚持跟踪服务，强化政策倾斜，打造一支会发展、敢担当、愿奉献、强引领的贫困村创业致富带头人队伍。

各位代表，脱贫攻坚已经到了啃硬骨头、攻坚拔寨的决胜阶段，我们将以更精准的举措、超常规的力度，向深度贫困发起总攻，坚决实现决战决胜！

（七）抓好污染防治攻坚，着力推动生态环境质量持续提升。把环境保护作为严肃的政治任务和重大民生工程，聚焦突出问题整改，以壮士断臂的决心坚决打好污染防治攻坚战。

一要建设更加天蓝水清的生态环境。严格落实常态督查、联合执法、考核问责、有奖举报等机制，积极争取中央、省生态环境保护专项资金支持，铁腕治污、依法治污、科学治污。坚决打赢蓝天保卫战。以明显降低细颗粒物浓度为重点，持续开展大气污染防治行动，确保今年市城区空气质量指数持续好转，SO2年均浓度持续下降。一是加强燃煤污染治理，继续扩大市城区和各县建成区禁燃区范围和清洁取暖覆盖率，持续推进燃煤锅炉整治，在继续坚持宜热则热、宜电则电、宜气则气的治理基础上，大力推广“以醇代煤”，重点在108国道、高铁沿线和市城区周边以及各县（区、市）重点区域推行；同时，将其作为产业来培育，按照“研发新高地、行业排头兵、产业集聚区”目标定位，用三年时间加快培育成具有先进制造水平的百亿产业集群，今年要基本建成装备制造和存储配送两个中心。二是推进1200余台工业炉窑综合整治和焦化企业升级达标，倒逼淘汰落后产能、腾出环境容量。三是强化区域联防联控，坚决完成汾渭平原秋冬季大气污染综合治理各项目标任务。四是坚决抓好中央环保督察和汾渭平原强化督察问题整改。五是强化重点区域综合治理，抓好大西高铁、108国道沿线特别是15个综合服务区环境整治。坚决打好碧水保卫战。围绕潇河水质稳定达Ⅲ类和汾河流域水质消除劣Ⅴ类目标，对汾河、潇河沿线14个污水处理厂全部实施保温提效或扩容建设，进一步落实对汾河、潇河及其支流沿河排污口集中整治措施，开展河道“清四乱”专项行动，在寿阳和市城区段启动污水净化工程；狠抓工业水污染防治，推进农业农村水污染防治，完成9个县级集中式地表水饮用水水源地整治。坚决打好净土保卫战。加快煤矸石、粉煤灰污染综合治理，出台全市工业固体废物污染防治规划，有序开展受污染地块治理修复，坚决减存量、遏增量。

二要打造更加坚实牢固的生态安全屏障。落实国土空间规划，尽快完成我市“三线一单”编制，严格把握生态功能区划分；全面开展国土绿化行动，着力推动省交控集团与寿阳合作的苗木基地建设，打造“集团公司+合作社+农户”的绿色产业航母，全市林木绿化率增长1个以上百分点；推进汾河流域生态修复治理，实施好介休龙凤河、平遥柳根河湿地、太谷东咸阳段修复。

三要形成更加集约节约的生产生活方式。全面落实节能降耗措施，推进重点用能单位能耗在线监测系统建设，完成装机容量30万千瓦以上火力发电厂的接入端系统建设，实现用能单位全能源品种能耗指标和企业能效指标在线采集。倡导绿色生活方式，增加绿色供给，扩大绿色建筑标准强制执行范围，大力推广节水家电和节能建材，促进居民绿色消费、低碳生活。

（八）抓好群众关心关注的热点难点问题，着力推动民生改善再上新台阶。始终坚持以人民为中心的发展思想，针对人民群众普遍关心的问题精准施策，让改革发展的成果更多更公平惠及全市人民，让人民群众更加增强对党的信心、信仰和信赖。

一要不断丰富公共服务供给。坚持教育优先发展。实施“百亿教育投资计划”，从今年起三年投资120亿元上马218个教育项目；整合引进优质教育资源，培养优秀人才，创造条件建设应用型本科院校；全面推行“县管校聘”改革，真正把办学自主权交给校长。加快健康晋中建设。持续推进医疗、医保、医药“三医联动”改革，建立分级诊疗、现代医院管理、药品供应保障等五项制度；探索搭建“互联网+医疗健康”平台，加强专业人才引进培养，加强基层医疗卫生机构标准化、规范化建设，提升诊疗能力和服务水平。促进文体事业繁荣发展。积极发挥各级文化场馆的作用，全面提升公共文化服务效能，深入开展文化惠民和文化下乡活动。认真办好二青会承办项目和第五届市运会，举办好首届晋中国际马拉松赛，完成2000公里健身步道建设。

二要做好就业和社会保障工作。始终把稳就业摆在突出位置，重点做好高校毕业生、农民工、退役军人、城镇就业困难人员等就业工作；深入开展失业保险援企稳岗“护航行动”，支持困难企业开展职工在岗培训；大力支持创业载体建设，推进大学生创业园等创业孵化示范基地建设。继续深化社保制度改革，进一步推进全市机关事业单位养老保险制度改革，做好全市自收自支事业单位养老保险参保，继续完善生育和医疗保险合并实施。加快养老服务体系建设，每千名老人床位达到39张。

三要精心维护社会安全稳定。牢固树立大安全、大应急理念，统筹推进安全生产领域改革，继续以“深化煤矿安全攻坚年活动”为重点，严字当头、清单管理、巡查考核、上限处罚，并在各行业领域比照煤矿安全问责办法，对工作不力导致事故或严重社会影响的，依法依规严肃问责，以此促进各行业领域解决突出问题，防范和遏制较大及以上生产安全事故发生，牢牢稳住安全生产基本盘。坚持“优化、协同、高效”原则，以推进精细管理、精准预警、精确应急为核心，进一步提升全市应急保障和处置能力，提高防灾减灾救灾水平。深化“平安晋中”建设，开展好“七五”普法教育，加强信访维稳，健全完善社会治安防控体系，推动扫黑除恶专项斗争向全面纵深开展。同时，要持续发展妇女儿童、老龄、慈善和红十字会等事业，增强做好民族宗教、外事、侨务、对台等工作的意识，支持国防建设，加强双拥工作，做好退役军人事务工作，开展好第四次全国经济普查。

各位代表，办好民生实事是政府最大的责任。今年我们在广泛征集民意的基础上，围绕群众最关心的重点领域安排了13件实事：一是继续实施全民技能提升工程，再培训5万多人；二是将残疾预防重点干预和残疾儿童抢救性康复项目帮助对象由3630名增加到4500名；三是继续为榆社、左权、和顺、昔阳4县农村妇女提供免费“两癌”检查服务；四是继续为怀孕妇女提供免费产前筛查与诊断服务；五是新建70个农村老年人日间照料中心；六是继续免费送戏下乡915场；七是实施经济困难的高龄和失能老年人关爱行动；八是实施免费法律咨询便民工程；九是再建设村级物流节点800个，实现千人以上建制村物流节点全覆盖；十是继续推进农村厕所无害化改造；十一是培训基层中医适宜技术人才1000名，提升基层中医药服务能力； 十二是开设晋中中小学课外公益辅导“空中课堂”，有效满足学生高质量课后学习需求；十三是将肉牛养殖保险覆盖范围扩大到和顺、昔阳、左权和榆社。我们就是要把惠及百姓的实事一件接着一件办，办一件成一件，让人民群众得到实实在在的获得感、幸福感！

三、加强政府自身建设

坚持以政治建设为统领，树牢“四个意识”，坚定“四个自信”，坚决做到“两个维护”，扎实开展“不忘初心、牢记使命”主题教育和“改革创新、奋发有为”大讨论，认真落实“三个三”工作思路和举措，对标一流、追求卓越，以恒久的坚韧和执着的毅力，努力建设人民满意的服务型政府。

一要严格依法行政，提升政府法治水平。全面推进依宪施政、依法行政，自觉接受市人大及其常委会的法律监督、工作监督和市政协的民主监督，认真办理人大代表建议和政协委员提案，广泛听取民主党派、无党派人士、工商联、人民团体和社会各界意见。接受监察监督和审计监督，支持开展司法监督，主动接受社会和舆论监督。做好《扬尘污染管理条例》《医疗垃圾管理条例》《城市街道管理条例》立法调研起草工作。加强行政执法规范化和行政执法监督。深化政务公开。

二要深化效能革命，提升政务服务水平。结合机构改革重点推进审批服务便民化，提升创新策源能力，在常态化运营“共享晋中10360”基础上，整合资源，建设集政务服务、城市管理、公共服务三大领域十大板块的“一网十平台”，统筹政务数据标准化管理和政务服务标准化建设，打造阳光政务和“不打烊”政府，提升政务服务便捷化、城市管理精细化、公共服务便利化水平。深入推进“照后减证”“准入即营”改革，解决企业“准入不准营”问题；推进“多证合一”，使营业执照成为企业唯一“身份证”。推进监管创新，用好“互联网+监管”平台，实行“三随机三公开”，促进营商环境持续优化，着力构建亲清新型政商关系。

三要发扬斗争精神，提升工作落实水平。扎实开展“改革创新、奋发有为”大讨论，在政府系统掀起一场学习的革命、思想的革命、工作的革命，促进理念大提升、本领大提升、作风大提升，提高推进工作的标准和质量，确保中央和省市重大决策部署落地见效。要扛起责任抓落实，各级主要领导要当好执行者和推动者，人要沉下去、事要担起来，一线传导压力，层层压实责任，推动落实到位。要学用政策重创新，努力提升专业素养，创造性贯彻落实上级大政方针和决策部署，注重从政策中找机遇、找项目、找资金、找方法，通过用好政策保障任务落实。要担当作为出实效，树立担当为荣、避事为耻的理念，碰到难题敢于触及，遇到矛盾主动化解，担在关键、干在实处、走在前列，既出力又出彩，以担当作为实际成效彰显能力水平，体现对党的忠诚。

四要全面从严治党，提升作风和廉政建设水平。落实全面从严治党主体责任，巩固发展反腐败斗争压倒性态势，管好用好公共资金，抓好重点领域监管，严格落实领导干部经济责任审计规定，严防群众身边不正之风和腐败问题。锲而不舍落实中央八项规定精神，大力整治不敬畏、不在乎、喊口号、装样子等错误行为，整治空泛表态、应景造势、敷衍塞责、出工不出力以及“重痕不重绩、留迹不留心”等形式主义、官僚主义突出问题。政府工作人员要廉洁修身、干净干事，惕厉自省、勇毅前行，绝不辜负人民公仆的称号。

各位代表，“每一个人都是新时代的见证者、开创者、建设者”。让我们更加紧密团结在以习近平同志为核心的党中央周围，高举习近平新时代中国特色社会主义思想伟大旗帜，在省委、省政府和市委的坚强领导下，用“奋斗”定义2019年，以不扬鞭、自奋蹄和昼无为、夜难寐的精神，锐意开拓，务实创新，全面拓展晋中高质量转型发展新局面，以优异成绩迎接共和国70华诞！
